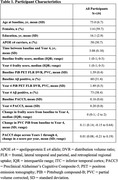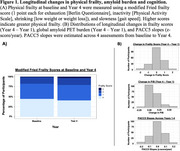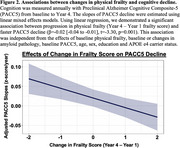# Longitudinal Increases in Physical Frailty are Linked to Accelerated Cognitive Decline, Independent of Preclinical Amyloid Pathology

**DOI:** 10.1002/alz70861_108668

**Published:** 2025-12-23

**Authors:** Paola A. Matos, Kailee A. Palmgren, Akpevweoghene P. Ikoba, Talia L. Robinson, Stephanie A. Schultz, Dylan R. Kirn, Michael J. Properzi, Aaron P. Schultz, Rachel F. Buckley, Rebecca E. Amariglio, Dorene M. Rentz, Keith A. Johnson, Reisa A. Sperling, Jasmeer P. Chhatwal, Wai‐Ying Wendy Yau

**Affiliations:** ^1^ Massachusetts General Hospital, Boston, MA USA; ^2^ Medical College of Wisconsin, Milwaukee, WI USA; ^3^ Brigham and Women's Hospital, Boston, MA USA; ^4^ Harvard Medical School, Boston, MA USA; ^5^ Athinoula A. Martinos Center for Biomedical Imaging, Charlestown, MA USA; ^6^ Center for Alzheimer Research and Treatment, Brigham and Women’s Hospital, Boston, MA USA

## Abstract

**Background:**

Physical frailty is a risk factor for dementia including Alzheimer’s Disease, representing a potentially modifiable target for prevention. However, the relationship between longitudinal changes in frailty and amyloid pathology in cognitively unimpaired older adults, and their joint effects on cognitive decline remains unclear.

**Method:**

We examined data from 191 cognitively unimpaired older adults in the Harvard Aging Brain Study with frailty measurements and amyloid PET (Pittsburgh Compound‐B) at baseline and Year 4, and annual cognitive assessments (Preclinical Alzheimer Cognitive Composite‐5; PACC5) (Table 1). Physical frailty was measured using a modified Fried frailty score (1 point each for exhaustion [Berlin Questionnaire], inactivity [Physical Activities Scale], shrinking [low weight or weight loss]), and slowness [gait speed]. We examined the associations between frailty score and amyloid burden at baseline, and across baseline and Year 4. We then estimated the slope of PACC5 decline across the four annual assessments using linear mixed effects models, and examined whether changes in frailty and/or amyloid predicted cognitive decline.

**Result:**

Physical frailty score and amyloid burden were not significantly associated at baseline (β=‐0.03, t=‐0.96, *p* =0.34), or across baseline and Year 4 (Baseline Amyloid∼Year 4 Frailty: β=0.01, t=0.04, *p* =0.97; Baseline Frailty∼Year 4 Amyloid: β=‐0.02, t=‐0.81, *p* =0.42), adjusting for age, sex, education and *APOE* e4. Higher baseline frailty (β=‐0.02, t=‐2.84, *p* =0.005) and longitudinal increase in frailty score (β=‐0.02, t=‐3.30, *p* =0.001) were independently associated with faster PACC5 decline across baseline to Year 4. These associations were further independent from the effects of baseline amyloid (β=‐0.06, t=‐3.86, *p* <0.001) or changes (β=‐0.03, t=‐0.64, *p* =0.53) in amyloid pathology, baseline PACC5 (β=‐0.01, t=‐0.53, *p* =0.60), or other covariates.

**Conclusion:**

Among clinically normal older adults, both baseline physical frailty and increases in physical frailty over three years were associated with accelerated cognitive decline during the same period. These associations were independent from the effects of baseline or longitudinal amyloid pathology, and baseline cognitive performance. These findings suggest that interventions targeting the prevention or reduction of physical frailty may help slow late‐life cognitive decline, independent of preclinical amyloid pathology, and may yield detectable effects within the typical timeframe of prevention trials.